# Evaluation of the effectiveness of hysteroscopic myomectomy in the symptoms of women with uterine myomatosis: a retrospective cohort

**DOI:** 10.1590/1806-9282.20241525

**Published:** 2025-06-02

**Authors:** Alana Baptista Fim, Cristina Laguna Benetti-Pinto, Daniela Angerame Yela

**Affiliations:** 1 Universidade Estadual de Campinas, School of Medical Sciences, Department of Gynecology and Obstetrics – Campinas (SP), Brazil.

**Keywords:** Myoma, Metrorrhagia, Hysteroscopy

## Abstract

**OBJECTIVE:**

The aim of this study was to evaluate the effectiveness of hysteroscopic myomectomy in the symptoms of women with uterine myomatosis.

**METHODS:**

This is a retrospective cohort study conducted on 119 women with uterine myomatosis who underwent hysteroscopic myomectomy in a tertiary hospital from 2018 to 2023. Women of reproductive age diagnosed with submucosal myoma who underwent hysteroscopic myomectomy were included, and women who did not have the data in their medical records necessary for completion were excluded. The sociodemographic and clinical variables of these women were evaluated.

**RESULTS:**

The average age of the women was 41.4±6.8 years. Among the fibroids, 37.0% were International Federation of Gynecology and Obstetrics (FIGO) 0 and 52.5% were FIGO 1. The average size of the fibroids was 2.7±1.4 cm. There were 10.0% of complications. After myomectomy, 67.2% of the women had improvement in symptoms, 66.1% opted for hormonal treatment, and 14.3% underwent hysterectomy. Ninety percent of the women who did not show improvement in symptoms had FIGO 1 and FIGO 2 fibroids (p=0.002), and 57% had a higher number of cesarean sections (p=0.038). In addition, 61% of these women required a new approach (p<0.001), and 93% opted for treatment after myomectomy (p<0.001). Factors associated with a greater chance of women's symptoms not improving were having more than two cesarean sections (hazard ratio [HR]=3.52, p=0.026), FIGO 1 fibroids (HR=5.75, p=0.003), and FIGO 2–3 fibroids (HR=8.25, p=0.030).

**CONCLUSION:**

Hysteroscopic myomectomy has a low complication rate, and having fibroids with a larger intramural component is the main factor responsible for myomectomy failure.

## INTRODUCTION

Among the various gynecological diseases studied, uterine myomatosis is of great relevance, being considered the most prevalent benign tumor in the uterus. Its origin occurs in the uterine muscle tissue itself, where myometrium cells present clonal expansions^1^. The exact prevalence of uterine myomatosis is not well established, as most studies evaluate symptomatic women. An American study showed a prevalence of myomatosis in more than 80% of Afro-descendant women and 70% of Caucasian women up to the age of 50 years^2^.

Its symptoms are variable, and women may be asymptomatic, with the disease detected through incidental findings on imaging tests. When symptomatic, they may present with abnormal uterine bleeding (AUB), pelvic pain, a feeling of heaviness and pressure in the pelvic region, infertility, and repeated miscarriages^3^. The clinical presentation is variable, depending on the location of the fibroid in relation to the uterus. Due to this variation, the International Federation of Gynecology and Obstetrics (FIGO) presented the classification into nine types of uterine fibroids according to their location (submucosal fibroids, FIGO 0–2; intramural fibroids, FIGO 3–4; subserous fibroids, FIGO 5–7; and other locations—cervical, round ligament, broad ligament, and parasite—FIGO 8)^4^. Among them, submucosal fibroids, due to changes in endometrial conformation, may present the highest rates of AUB and consequently anemia^3^.

The clinical consequences of anemia are already well established; however, in addition to these, women undergoing specific treatments have worse outcomes when they have anemia. They have longer hospitalization times and higher rates of intraoperative and postoperative complications^3^. Furthermore, anemia is considered one of the main reasons for indicating hysterectomy^5^, and adequate treatment of submucosal fibroids, for example, hysteroscopy, could reduce the number of unnecessary hysterectomies^6^.

Uterine myomatosis is present in up to 10% of women accompanied by infertility and can be cited as the sole cause in 1–2.4% of women^7^. Fibroids can distort the uterine cavity, with endometrial changes that do not allow the pregnancy to develop properly. The management of uterine myomatosis seeks to control the woman's symptoms. The available treatments allow better control of symptoms, especially the symptoms of AUB. Clinical treatment allows the use of non-steroidal anti-inflammatory drugs, tranexamic acid, combined hormonal oral contraceptives or progestins, and gonadotropin-releasing hormone agonists or antagonists^1^. Other available treatments can be mentioned, such as uterine artery embolization and resonance-guided radiofrequency ablation.

Surgical management includes options, such as myomectomy, hysterectomy, and endometrial ablation. Hysterectomy is considered the only definitive treatment option; however, we do not use it if the patient does not have complete offspring. Myomectomy can be performed in different ways, such as laparoscopic, laparotomic, and hysteroscopic. The hysteroscopic approach is indicated for smaller and submucosal fibroids.

The hysteroscopic approach has high success rates, between 70 and 99%^8^. Despite being considered one of the most complex hysteroscopic surgeries, there is safety and efficacy in the treatment of submucosal fibroids, making it an excellent therapeutic option in these cases. Hysteroscopic resection of fibroids poses a risk of minor complications, with faster recovery, and could allow obstetric delivery^9^. The literature on the effect of surgical hysteroscopy and fertility is still inconsistent and requires more robust evidence. This study aimed to evaluate the effectiveness of hysteroscopic myomectomy in the symptoms of women with uterine myomatosis.

## METHODS

This is a retrospective cohort study in which 119 women who underwent hysteroscopic myomectomy were analyzed from January 2018 to December 2022 in a tertiary hospital. Only women of reproductive age between 18 and 50 years with a diagnosis of submucosal myoma by ultrasound who underwent hysteroscopic myomectomy were included, and women who did not have the data in their medical records necessary for completion were excluded.

Data collection was carried out through analysis of these women's electronic medical records, which were organized in an Excel spreadsheet. The variables analyzed were age; skin color (white and non-white); marital status (with and without a partner); parity; smoking (yes and no); body mass index (BMI—calculated by dividing the weight in kilograms by the square of the height in meters); AUB (yes and no); symptom time (AUB—months); comorbidities (high blood pressure, diabetes mellitus, depression, hypothyroidism, and others); previous surgeries (cesarean section, laparotomy, laparoscopy, and others); year of myomectomy (2018–2019 and 2020–2022); myoma classification according to the classification of FIGO (FIGO 0—totally intracavitary, FIGO 1—where more than 50% is intracavitary, FIGO 2—where more than 50% is intramural, FIGO 3—intramural touching the endometrium and cervical myoma—FIGO 8); myoma size (centimeters); treatment prior to hysteroscopy (none; progestin alone—pill; intramuscular, intrauterine, and subdermal; and combined oral hormonal contraceptives); infertility (yes and no); reproductive desire (yes and no); dyspareunia (yes and no); dysmenorrhea (yes and no); anemia (characterized by the presence of hemoglobin below 11 g/dL), hemoglobin (considered normal values above 11 g/dL), and hematocrit (considered normal values above 36%), which were evaluated before and after the procedure; complications (overload, uterine perforation, partial myoma resection, and bleeding); treatment after the procedure (none; progestin alone—pill; intramuscular, intrauterine, and subdermal; and combined oral hormonal contraceptives); hysterectomy (yes and no); and improvement in symptoms after the procedure (yes, no, partial, and lost to follow-up).

This research was approved by the institution's Research Ethics Committee (approved on September 6, 2023, under number: 6,285,404; CAAE: 71004723.0.0000.5404).

### Statistical analysis

The frequency, means, and standard deviation of the variables were calculated. Fisher's exact test or the chi-square test was used to compare categorical variables. To compare numerical variables, the Mann-Whitney test was used due to the absence of normal distribution. Cox regression analysis was used to assess the factors related to improvement in women's clinical symptoms by calculating the hazard ratio (HR) with a 95% confidence interval (CI). A significance level of 5% was used, and SAS version 9.4 (Cary, NC) was used for the statistical analysis.

## RESULTS

**Figure 1:**
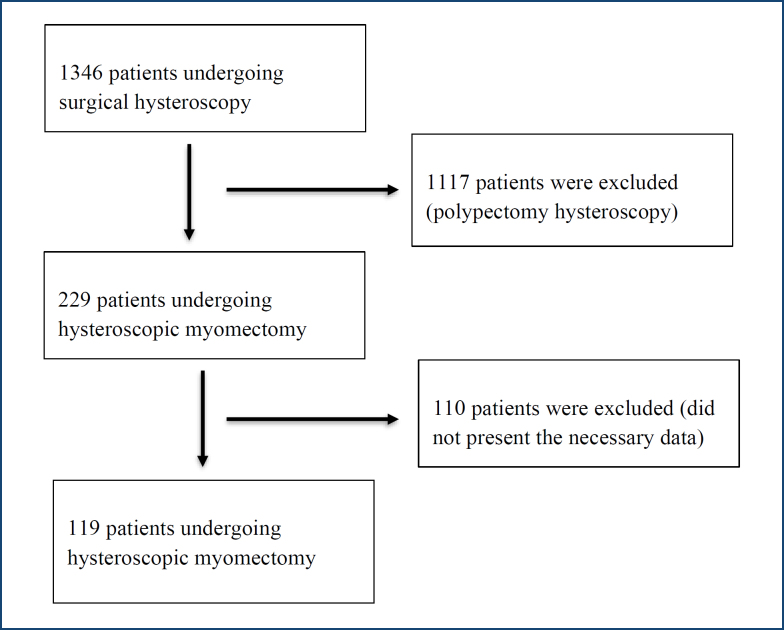
Flowchart of participant selection.

A total of 1,346 medical records of women who underwent surgical hysteroscopy were analyzed. Of these, 229 were hysteroscopic myomectomy. Exclusions were made for 110 women because they did not present the necessary data for the research. Thus, 119 women who underwent hysteroscopic myomectomy were analyzed (Figure 1).

Of the women selected (119), who underwent surgical hysteroscopy for submucosal myoma, 86.5% were aged between 30 and 49 years, with an average of 41.4±6.8 years, the majority (71.4%) had a white skin color, already had at least one child, 49.6% had at least one previous cesarean section, and 40.4% had no surgeries. The average BMI was 29.6±6.3 kg/m^2^. The most prevalent comorbidity was high blood pressure (25.2%) (Table 1).

**Table 1:** Sociodemographic, clinical, and surgical characteristics of women with uterine fibroids undergoing hysteroscopic myomectomy (n=119).

Variable		Mean±SD/n (%)
Age (years)		41.4±6.8
Nulligravid		24 (22.0)
White skin color		85 (71.4)
Living with a partner		33 (61.1)
BMI (kg/m^2^)		29.6±6.3
Smoking		6 (8.4)
Without previous surgeries		36 (40.4)
Comorbidities		55 (46.2)
Year of treatment		
	2018–2019	55 (46.2)
	2020–2022	64 (53.8)
Type of myoma		
	FIGO 0	43 (37.0)
	FIGO 1	61 (52.5)
	FIGO 2	5 (4.3)
	FIGO 3	1 (0.8)
	FIGO 8	6 (5.1)
AUB		117 (98.2)
Infertility		5 (13.8)
Anemia		31 (26.5)
Use of HT before myomectomy		92 (76.6)
Complications		12 (10.0)
Desire to undergo another surgery		37 (31.0)
Pre-surgery hemoglobin (g/dL)		12.1±2.1
Hemoglobin after surgery (g/dL)		11.6±2.0
Use of HT after myomectomy		81 (66.1)
Lost to follow-up		12 (10.0)

SD: standard deviation; BMI: body mass index; FIGO: International Federation of Gynecology and Obstetrics, AUB: abnormal uterine bleeding, HT: hormonal therapy.

Of the fibroids, 37.0% were FIGO 0, 52.5% were FIGO 1, and 5.1% were delivered. The average size of the fibroids was 2.7±1.4 cm, with the smallest being 0.5 cm and the largest 6 cm. Of the complaints reported, 98.3% had AUB and the average time was 24.2±23.4 months, with 26.5% of women having some degree of anemia resulting from this bleeding. The majority of women received some kind of previous treatment (76.5%), with oral progestin pills being the most used (33.9%) (Table 1).

Of the 119 hysteroscopies performed, only 12 had complications, including 5 uterine perforations, 2 overload, and 5 partial myoma resections. Notably, 31% of the women needed a new approach. After myomectomy, 67.2% of the women had improvement in symptoms, 66.0% opted for hormonal treatment (progestin pills and combined oral hormonal contraceptives), and 14.3% underwent hysterectomy (Table 1).

Women who did not show improvement in symptoms had myoma FIGO 1 and FIGO 2 (p=0.002) and more than one cesarean section (p=0.038). Furthermore, these women expressed their desire to undergo another surgery (p<0.001), and a greater number of women opted for treatment after myomectomy (p<0.001) (Table 2).

**Table 2:** Clinical characteristics of women with uterine fibroids undergoing hysteroscopic myomectomy according to clinical improvement (n=107).

	With improvement (n=76), mean±SD/n (%)	Without improvement (n=31), mean±SD/n (%)	p
Age (years)	41.8±6.8	41.3±7.1	0.623
Nulligravid	18 (25.7)	6 (20.0)	0.387
White skin color	55 (72.3)	23 (74.1)	0.964
BMI (kg/m^2^)	29.1±6.4	31.3±6.0	0.079
Cesarean section ≥1	31 (44.8)	16 (57.1)	0.038
Type of myoma			0.002
FIGO 0	33 (44.5)	4 (13.3)	
FIGO 1	33 (44.5)	23 (76.7)	
FIGO 2	2 (2.7)	3 (10.0)	
FIGO 3	1 (1.3)	0	
FIGO 8	5 (6.7)	0	
Size of myoma (cm)	2.6±1.3	2.8±1.6	0.768
AUB	76 (100.0)	30 (96.7)	0.290
Symptom time (AUB—months)	22.0±18.3	30.7±32.6	0.368
Desire to undergo another surgery	15 (19.7)	19 (61.2)	<0.001
Pre-surgery hemoglobin (g/dL)	12.2±2.3	11.9±1.7	0.138
Hemoglobin after surgery (g/dL)	11.0±2.3	12.3±1.5	0.194
Treatment after myomectomy	45 (59.2)	29 (93.5)	<0.001

SD: standard deviation; BMI: body mass index; FIGO: International Federation of Gynecology and Obstetrics, AUB: abnormal uterine bleeding.

The factors associated with a greater chance of no improvement in women's symptoms were having more than two cesarean sections (HR: 3.52, 95%CI 1.11–10.68; p=0.026), myoma FIGO 1 (HR: 5.75, 95%CI 1.79–18.46; p=0.003), and myoma FIGO 2–3 (HR: 8.25, 95%CI 1.23–55.56; p=0.030) (Table 3).

**Table 3:** Risk factors associated with non-improvement in women with uterine fibroids undergoing hysteroscopic myomectomy (n=107).

		Crude HR	HR with a 95%CI	p	Adjusted HR*	Adjusted HR with a 95%CI*	p
Age (years)		0.990	0.932–1.051	0.739			
White skin color		0.91	0.35–2.35	0.847			
With a partner		1.60	0.40–6.47	0.510			
Gestation							
	1	0.83	0.22–3.23	0.792			
	≥2	1.68	0.57–4.94	0.349			
Cesarean section							
	1	0.86	0.28–2.63	0.796			
	≥2	3.52	1.16–10.68	0.026	5.50	1.60–18.91	0.007
	Smoking	0.37	0.04–3.39	0.380			
	BMI (kg/m^2^)	1.054	0.986–1.127	0.124			
	Previous surgeries	0.65	0.26–1.64	0.357			
	Symptom time (AUB—months)	1.014	0.995–1.035	0.153			
	Comorbidities	0.78	0.34–1.83	0.574			
	Size of myoma (cm)	1.079	0.803–1.451	0.612			
Type of myoma (FIGO)							
	1	5.75	1.79–18.46	0.003			
	2 or 3	8.25	1.23–55.56	0.030			
	8	0.68	0.03–14.40	0.445			
	AUB	7.52	0.30–189.84	0.117			
	Previous treatments	2.23	0.69–7.21	0.180			
	Infertility	0.39	0.04–3.93	0.422			
	Reproductive desire	3.40	0.98–11.78	0.054			
	Anemia	1.21	0.47–3.07	0.695			
	Desire to undergo another surgery	7.02	2.76–17.86	<0.001	5.34	1.82–15.68	0.002
	Treatment after myomectomy	9.99	2.22–44.95	0.003	5.03	1.01–25.01	0.048

HR: hazard ratio; 95%CI: confidence interval; AUB: abnormal uterine bleeding; FIGO: International Federation of Gynecology and Obstetrics; BMI: body mass index.

* Stepwise criteria for selection of variables.

## DISCUSSION

In our study, most women had AUB. Among the myomas, FIGO 1 was the most prevalent. Most women had received some prior treatment. There were 10% of complications. After myomectomy, most women showed improvement in symptoms. Women who did not show improvement in symptoms had FIGO 1 and 2 myomas and a higher number of cesarean sections and required more surgical approaches, and a higher number of women opted for treatment after myomectomy. The factors associated with a higher chance of no improvement in women's symptoms were having more than two cesarean sections and FIGO 1 and FIGO 2–3 myomas.

Uterine myomatosis is one of the most common causes of AUB. Submucosal fibroids account for about 5.5–10% of all fibroids and are more related to AUB and infertility due to the increase in the endometrial area and distortion of the uterine cavity^1,10^.

Its prevalence varies greatly depending on the location and population studied. Other risk factors are obesity, nulliparity, family history, advanced age, and smoking^11^. The majority of women in this study had a white skin color, with some degree of overweight, and were over 30 years old, characteristics compatible with the epidemiology of myomatosis, except that the majority had some previous pregnancy. Although myomatosis is more related to people of African descent, studies show a high prevalence also in the light-skinned population. A study in the United States showed that they are detected on imaging tests regardless of symptoms in more than 80% of women of African descent and in 70% of light-skinned women at age 50 years^1^.

The majority of submucosal fibroids found in hysteroscopies were FIGO 0 and 1, fibroids with more submucosal than intramural components, and their average size was 2.74 cm. These factors are important to evaluate the success rate of fibroid resection and the chance of a new approach. STEP-W is a system that evaluates a set of factors and allows a standardized description of the fibroid to evaluate the best approach depending on the punctuation. These factors include size, topography, extension of the base in relation to the uterine wall, penetration into the myometrium, and whether it is located on the lateral wall or not. From this, a fibroid classified from 0 to 4 is considered of low complexity, 5 to 6 is considered of high complexity, and 7 to 9 is considered another technique other than hysteroscopy^12^.

According to the most recent guideline from the International Society for Gynecologic Endoscopy (ISGE), aiming for greater safety of the procedure, the endometrial cavity should be evaluated with ultrasound and diagnostic hysteroscopy, and then STEP-W should be performed^13^. In this study, submucosal fibroids with a myometrial component (FIGO 1 and FIGO 2) were related to no clinical improvement after the procedure, which may be related to the fact that these fibroids are more difficult to be completely resected.

Hysteroscopy, both outpatient and surgical, is considered a safe procedure with low complication rates. In a review that evaluated more than 11,000 diagnostic and 2,500 surgical hysteroscopies, it was concluded that surgical hysteroscopy has higher rates of complications, of which 0.2% were overload (especially in myomectomies) and 0.76% were uterine perforations (70% of perforations were at the time of dilation)^14^. In another study in which more than 21,000 surgical hysteroscopies were analyzed, the complication rate was 0.22%. The most common complication was also perforation of the uterus (0.12%), followed by fluid overload (0.06%), intraoperative hemorrhage (0.03%), and bladder or bowel injury (0.02%)^15^.

The complication rate for the procedures analyzed in this study was 10%, including uterine perforation, overload, and partial resection. These numbers are higher than those described in the literature, probably because hysteroscopies performed by gynecologists in training and with less experience were analyzed, increasing the risk of complications^8^.

In the literature, women undergoing hysteroscopic myomectomy have a bleeding improvement rate of 70–99%. The risk factors identified for failure were the number of fibroids, fibroid size, uterine size, and incomplete surgery^16^. In this study, the rate of improvement in post-procedural symptoms was 67.2%, and there was a statistically significant improvement in post-procedural hemoglobin and hematocrit rates. Risk factors for non-improvement and the need for a new surgical approach and treatment after the procedure were considered. Some submucosal fibroids require a new approach usually because of some complications in the first approach, or due to the size and position of the fibroid, which makes the procedure and its removal difficult, occasionally requiring another procedure for complete resection^8^.

Another factor that was related to a greater risk of no clinical improvement with statistical significance was the history of two or more cesarean sections. With the significant increase in cesarean section rates (Brazil has the second highest cesarean section rate in the world), the number of isthmocele cases is increasing. This condition is characterized by a defect in the uterine wall caused by a previous uterine scar that causes myometrial discontinuity and can cause AUB. Despite there being more and more studies on the subject, it is still underdiagnosed^17,18^.

The mechanisms involved in AUB due to isthmocele include blood retention in the defect, blood production through neovascularization, inflammation and adenomyosis, and fibrotic tissue at the site that impairs drainage^19^.

The main limitation of this study was the fact that it was a retrospective study with a lack of information on the consultations performed since the study was carried out through a review of medical records. Therefore, we cannot quantify AUB. In addition, due to the loss of information, many women could not be included, which resulted in a reduced sample size.

## CONCLUSION

Hysteroscopic myomectomy has a low complication rate and improves bleeding symptoms in most women. Having fibroids with a larger intramural component is the main factor responsible for myomectomy failure.
